# Characterization of the *Drosophila* BEAF-32A and BEAF-32B Insulator Proteins

**DOI:** 10.1371/journal.pone.0162906

**Published:** 2016-09-13

**Authors:** S. V. Satya Prakash Avva, Craig M. Hart

**Affiliations:** Department of Biological Sciences, Louisiana State University, Baton Rouge, Louisiana, United States of America; Institut de Genetique et Developpement de Rennes, FRANCE

## Abstract

Data implicate the *Drosophila* 32 kDa Boundary Element-Associated Factors BEAF-32A and BEAF-32B in both chromatin domain insulator element function and promoter function. They might also function as an epigenetic memory by remaining bound to mitotic chromosomes. Both proteins are made from the same gene. They differ in their N-terminal 80 amino acids, which contain single DNA-binding BED fingers. The remaining 200 amino acids are identical in the two proteins. The structure and function of the middle region of 120 amino acids is unknown, while the C-terminal region of 80 amino acids has a putative leucine zipper and a BESS domain and mediates BEAF-BEAF interactions. Here we report a further characterization of BEAF. We show that the BESS domain alone is sufficient to mediate BEAF-BEAF interactions, although the presence of the putative leucine zipper on at least one protein strengthens the interactions. BEAF-32B is sufficient to rescue a null *BEAF* mutation in flies. Using mutant *BEAF-32B* rescue transgenes, we show that the middle region and the BESS domain are essential. In contrast, the last 40 amino acids of the middle region, which is poorly conserved among *Drosophila* species, is dispensable. Deleting the putative leucine zipper results in a hypomorphic mutant BEAF-32B protein. Finally, we document the dynamics of BEAF-32A-EGFP and BEAF-32B-mRFP during mitosis in embryos. A subpopulation of both proteins appears to remain on mitotic chromosomes and also on the mitotic spindle, while much of the fluorescence is dispersed during mitosis. Differences in the dynamics of the two proteins are observed in syncytial embryos, and both proteins show differences between syncytial and later embryos. This characterization of BEAF lays a foundation for future studies into molecular mechanisms of BEAF function.

## Introduction

Like enhancers and promoters, insulators or boundary elements are a specialized class of regulatory DNA sequences. Insulators are defined by their ability to function in transgene assays to limit enhancer-promoter communication when placed between a promoter and an enhancer [1, 2], and to protect bracketed transgenes from chromosomal position effects [3, 4]. Based on these activities, it is thought that insulators separate genes into domains such that intra-domain regulatory interactions are preferred [5, 6] and spreading heterochromatin can be prevented from entering a domain [7–9]. Models propose that insulators demarcate the ends of chromatin domains by interacting to organize domains into loops, and these loops play an important role in controlling enhancer-promoter interactions and chromatin packaging [10, 11]. Although details of molecular mechanisms are unclear, support for these models comes from chromosome conformation capture experiments that have found that genomes are divided into topologically associating domains (TADs) that often have insulator binding proteins located at TAD boundaries [12–14].

Two of the first insulator elements identified are the *scs* and *scs'* sequences which bracket two *Hsp70* genes at the *87A* locus of *Drosophila* [15]. BEAF was identified as a Boundary Element-Associated Factor that binds to the *scs’* insulator, and was subsequently shown to immunolocalize to hundreds of interbands and band/interband boundaries of polytene chromosomes [16]. In addition to *scs’*, other genomic BEAF binding sites were shown to possess insulator activity [17, 18]. This implies that BEAF-associated insulators are common. BEAF is a complex of two 32 kDa proteins, named BEAF-32A and BEAF-32B (hereafter referred to as 32A and 32B, or collectively as BEAF) [19]. These two proteins are produced from the same gene, presumably by alternative promoters, and differ only in their N-termini of around 80 amino acids. Since both N-termini contain a single atypical DNA-binding zinc finger, called a BED finger [20], they bind different DNA sequences. The remaining approximately 200 amino acids are identical. The structure and function of the first 120 amino acids of this region is unknown, although it has been reported that it can be glycosylated and phosphorylated and may mediate association with the nuclear matrix [21]. The C-terminal region of around 80 amino acids mediates interactions between BEAF subunits and contains a BESS domain [22] and a putative leucine zipper. Interactions mediated by this region apparently can form trimers, and the ratio of 32A to 32B can vary at different loci on polytene chromosomes as observed by immunofluorescence [19].

Results using a dominant negative *BEAF* transgene or a null *BEAF* mutation showed that BEAF is essential for *scs’* insulator activity [23, 24]. This work also found that BEAF affects chromatin structure, is important for oogenesis and embryogenesis, and that 32B is essential while 32A is not. Maternally provided BEAF is sufficient for development to the adult stage, although females that cannot produce their own 32B are nearly sterile. Other data provide an unexpected link between BEAF and transcriptional regulation. Expressing a dominant negative *BEAF* transgene in eyes leads to a rough eye phenotype. Using this in a screen, genetic interactions between BEAF and 17 proteins were found. Most of these proteins are transcription factors or general transcription factors [25]. Interestingly, genomic mapping of BEAF binding found that 85% of 1820 BEAF peaks had their centers within 300 bp of a transcription start site (TSS) [26]. Other genome-wide studies found a similar correlation [27, 28]. Over 85% of the genes with a TSS within 300 bp of the center of a BEAF peak are on a list of housekeeping genes [29]. Together, these results suggest that BEAF may play a role at promoters to help maintain an architecture conducive to transcription, and perhaps interactions with transcription factors are involved. How this fits with traditional models of insulator function, and with data showing BEAF is an insulator protein that affects chromatin structure or dynamics, is presently unclear.

Here we report a further characterization of BEAF. We determine what region of BEAF mediates BEAF-BEAF interactions, and identify essential domains by testing the ability of 32B deletion mutants to rescue a null *BEAF* mutation. Because BEAF has been reported to remain on mitotic chromosomes [30] where it might serve as an epigenetic memory[31], we also characterize the dynamics of 32A and 32B tagged with fluorescent proteins during mitosis in embryos. The dynamics are more complex than expected, with differences observed between 32A and 32B as well as between syncytial and later embryos. They also provide a first indication that, in addition to playing a role on chromosomes, BEAF might play a role as a component of the mitotic spindle matrix, which is consistent with its reported physical interaction with Chromator [32, 33].

## Materials and Methods

### Plasmids and DNA

#### Yeast 2-Hybrid plasmids

**Fig 1:**
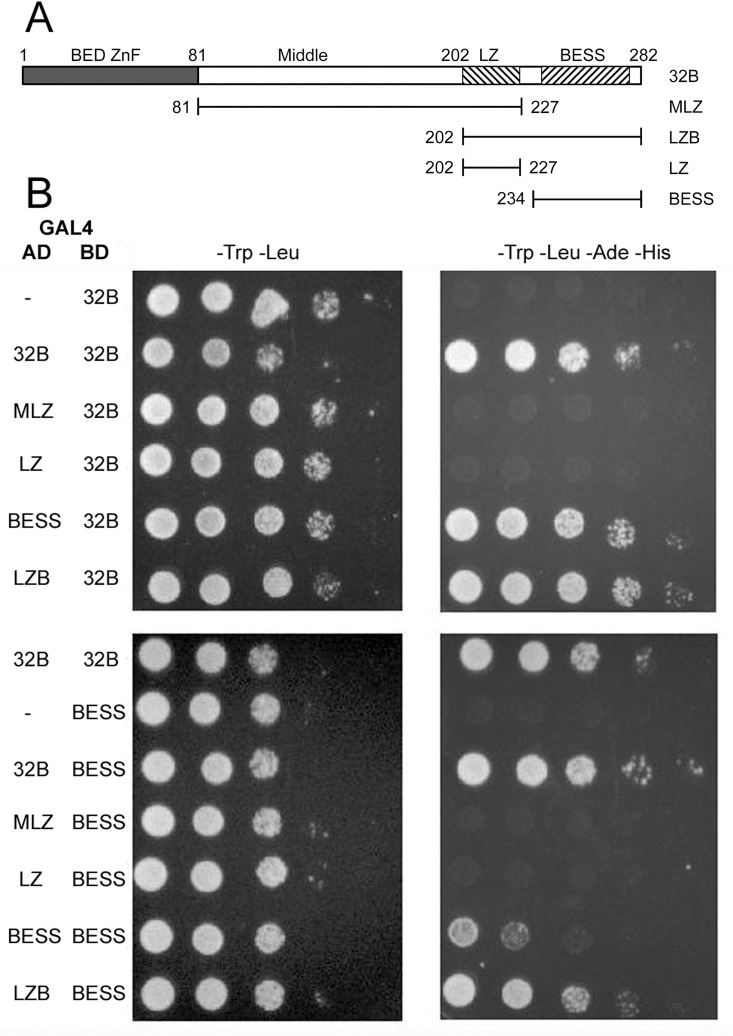
Yeast 2-hybrid assays demonstrate that BEAF-BEAF interactions are mediated by the BESS domain, and are strengthened if at least one protein also has the putative leucine zipper. A. Schematic of 32B and parts derived from 32B that were fused at the carboxy ends of the GAL4 DNA-binding domain (BD) and activation domain (AD). Gray rectangle: 32B unique sequences, which encompass the DNA-binding BED finger. First hatched rectangle: putative leucine zipper. Second hatched rectangle: BESS domain. Numbers indicate the first and last amino acid present in the truncated proteins. B. Representative Y2H results, with either 32B (top panels) or the BESS domain (bottom panels) fused to the GAL4 BD. Serial 10-fold dilutions of yeast were spotted onto the plates. Left panels (-Trp -Leu) show growth on plates selecting for the presence of the BD and AD plasmids. Right panels (-Trp -Leu -Ade -His) show growth on plates selecting for the presence of the BD and AD plasmids and the expression of two reporter genes. Reporter gene expression requires that both the BD and the AD have the BESS domain (bottom right panel, BESS BESS). More vigorous growth, similar to 32B 32B and LZB 32B, was obtained if at least one protein also had the putative leucine zipper (bottom right panel, 32B BESS and LZB BESS). Similar results were obtained when the BD and AD were switched (Table 1). Results not shown here are shown in S1 Fig.

Primers were designed so that sequences encoding 32B or parts of BEAF could be PCR amplified, cut with *Eco*RI and *Sal*I, and ligated in the correct reading frame to sequences encoding an N-terminal GAL4 activation domain in pOAD or GAL4 DNA binding domain in pOBD2 [34]. Fig 1A indicates the BEAF amino acids encoded in the MLZ, LZ, LZB and BESS constructs.

**Table 1:** Results of testing for interactions between parts of BEAF in Y2H assays.

	AD					
BD	-	32B	MLZ	LZ	BESS	LZB
-	-	-	-	-	-	-
32B	-	+	-	-	+	+
MLZ	-	-	-	-	-	-
LZ	-	-	-	-	-	-
BESS	-	+	-	-	+(w)	+
LZB	-	+	-	-	+	+

The GAL4 DNA binding domain (BD) or activation domain (AD) was located N-terminal of full-length 32B or the indicated parts of BEAF (Fig 1A). Plus indicates yeast growth (physical interaction); minus indicates no growth (no interaction); (w) indicates weak growth.

#### Pull-down plasmids

**Fig 2:**
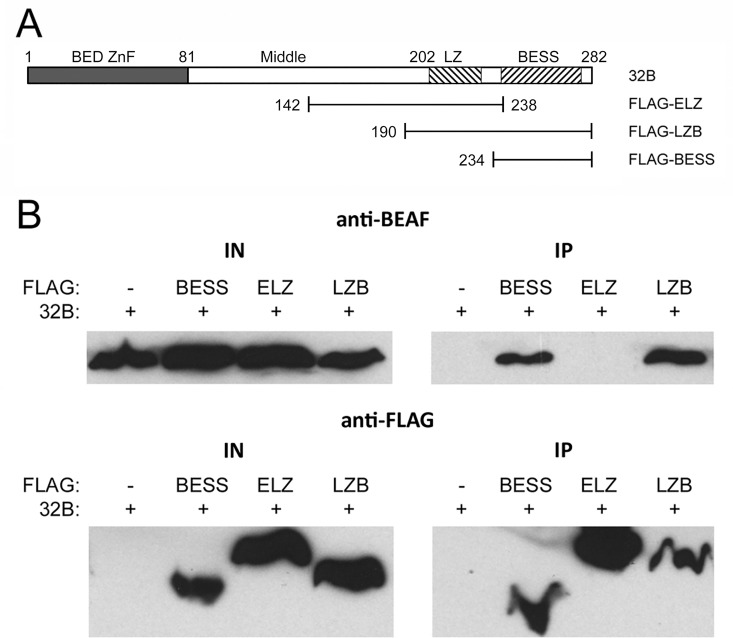
Pull-down experiments demonstrate that the BESS domain is sufficient for BEAF-BEAF interactions. A. Schematic of 32B, indicating the parts derived from 32B that had an N-terminal FLAG tag. Numbers indicate the first and last amino acid present in the truncated proteins. B. Western blot analysis of pull-down experiments. Proteins were co-expressed in *E*. *coli* and immunoprecipitated with antibodies directed against the FLAG epitope. Left panels show input proteins and right panels show immunoprecipitated proteins detected with anti-BEAF (top panels) or anti-FLAG (bottom panels) antibodies. Pull-down of 32B is only observed if the FLAG-tagged protein has a BESS domain (upper right panel).

The pET-32B plasmid [19] was modified by insertion of *Nsi*I and *Eco*RI cut PCR products containing a Shine-Dalgarno sequence followed by sequences encoding FLAG-tagged parts of BEAF to make bicistronic expression vectors. Also, sequences encoding the putative leucine zipper were extended to produce FLAG-ELZ (Extended Leucine Zipper) because we could not detect production of FLAG-LZ. Fig 2A indicates the BEAF amino acids present in the FLAG-ELZ, -LZB and -BESS constructs.

#### P-element plasmids

**Fig 3:**
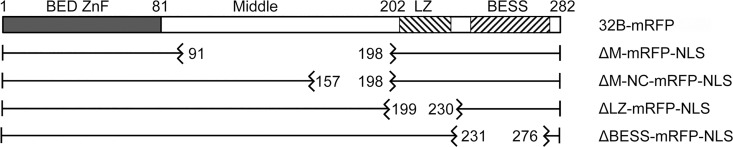
Schematic of the mutant 32B proteins tested for the ability to rescue a null *BEAF* mutation. Numbers indicate the first and last amino acids of the deleted segment. All deletion proteins had mRFP and an SV40 NLS fused at their carboxy ends, and transgene expression was driven by endogenous *BEAF* sequences as described in Materials and Methods.

Construction of a *P*-element plasmid encoding a *BEAF-EGFP* fusion gene driven by the endogenous *BEAF* promoter has been described [23]. The transgene is insulated upstream by the M2 derivative of the *scs’* insulator [18], and downstream of mini-*w* by the *scs* insulator [3]. About 900 bp of sequences upstream of the *32A* ATG start codon are present, from an *Eco*RI site about 260 bp upstream of the transcription start site of the divergently transcribed *CG10155* gene. To make a *32A-EGFP* transgene, site-directed mutagenesis was performed to introduce *Sa*lI sites about 40 bp upstream and 150 bp downstream of the *32B* start codon (Quikchange, Agilent Technologies) followed by deletion of the *Sal*I fragment. To make a *32B-mRFP* transgene, a *Bsi*WI site was placed about 30 bp upstream of the *32A* start codon by site-directed mutagenesis, and a *Bsi*WI fragment from this site to about 140 bp downstream of the start codon was deleted. Then sequences encoding a monomeric RFP protein [35] were PCR-amplified and substituted for *EGFP* sequences as a *Kpn*I-*Not*I fragment. This *32B-mRFP* gene fusion was also used to make transgenes lacking coding sequences for parts of BEAF. First an SV40 nuclear localization signal was placed at the carboxy end of mRFP. Then Quikchange site-directed mutagenesis was used to remove sequences coding for the amino acids indicated in Fig 3 for ΔM, ΔM-NC, ΔLZ and ΔBESS. All plasmids were verified by sequencing.

### Yeast 2-Hybrid

The Y2H assay was performed with plasmids, yeast strains, and protocols derived from the Fields lab [34, 36]. The GAL4 DNA binding domain bait plasmid pOAD has the *TRP1* gene, while the GAL4 activation domain prey plasmid pOBD2 has the *LEU2* gene. Plasmids were transformed into yeast strain Y2H Gold (Clontech) by the lithium acetate method. Co-transformants were selected on minimal medium lacking tryptophan and leucine (2-drop). After incubation for 3–4 days at 30°C, 8 single colonies were picked and streaked onto 2-drop and 4-drop (2-drop medium additionally lacking adenine and histidine) plates to test for activation of the *ADE2* and *HIS3* reporter genes by interactions between the bait and prey proteins. One colony was then grown in liquid 2-drop medium, diluted to an OD600 of 0.1, and 10-fold serial dilutions were spotted onto 2-drop and 4-drop plates.

### Protein expression in *E*. *coli* and immunoprecipitation

Proteins were expressed in the *E*. *coli* strain BL21 containing pLysS and protein extracts were prepared by standard methods [37]. 32B and FLAG-tagged parts of BEAF were co-expressed using a bicistronic strategy after interactions were not detected when they were expressed individually and extracts were mixed. For pull-downs, anti-FLAG M2 agarose beads (Sigma Aldrich) were rotated at room temperature for 30 minutes in blocking buffer (10mM Tris pH 8.0, 1 mM EDTA, 50 mM NaCl, 0.5 mM PMSF, 0.25% BSA). Then 5 μl of beads were mixed with 80 μl protein extract (10 mM Tris pH 8.0, 1 mM EDTA, 150 mM NaCl, 10% glycerol, 0.5 mM PMSF containing protease inhibitor cocktail (Sigma-Aldrich)), brought to a final volume of 400 μl using TEN.50 (10 mM Tris pH 8.0, 1 mM EDTA, 50 mM NaCl, 0.5 mM PMSF), and rotated for 2 hours at room temperature. The beads were then washed six times with 500 μl TEN.50 buffer, resuspended in 30 μl of 1x sample loading buffer, and heated to 95°C. Before the sixth wash, the beads were transferred to a new tube. Samples were split, loaded onto 2 gels together with input proteins, and separated by SDS-PAGE. Proteins were transferred to nitrocellulose membranes, probed with anti-BEAF [16] and anti-FLAG M2 antibodies (Sigma Aldrich) at 1:10,000 and 1:2,500 respectively, and detected with anti-rabbit HRP (BioRad) or anti-mouse-HRP (Jackson ImmunoResearch) at 1:10,000 using an enhanced chemiluminescence kit (Amersham).

### *Drosophila* stocks

P-element plasmids were sent to GenetiVision (Houston, TX) for generation of transgenic flies by microinjection into preblastoderm *y*^*1*^ *w*^*67c23*^ embryos. Stocks with *H2Av-EGFP* or *H2Av-mRFP* transgenes were obtained from the Bloomington *Drosophila* Stock Center (BDSC 23650 and 24163). Flies were maintained on standard cornmeal, yeast, and sugar medium with Tegosept and crosses were performed at 25°C.

### Ovary dissection and staining

Wild-type or *mutant*-*32B*; *BEAF*^*AB-KO*^ females were mated with wild-type males for 3 days before ovaries were dissected. Ovaries were dissected in PBS (137 mM NaCl, 2.7 mM KCl, 10 mM Na_2_HPO_4_, 1.8 mM KH_2_PO_4_, pH.7.4) and fixed with 3.7% formaldehyde in PBS for 2 minutes. The ovaries were then transferred to PBS with 0.1%Triton-X100, 1 μg/ml DAPI and stained for 2 min. Then the ovaries were washed in PBS for 2 min and placed on a glass slide with a drop of 50% glycerol in PBS and observed with a Zeiss Axioskop microscope equipped with a SPOT RT Slider CCD camera (Diagnostics Instruments).

### Time-lapse microscopy

Embryos of the desired genotype were dechorionated, placed on a coverslip attached to a Plexiglas slide with a 1 cm hole in the center, and covered with halogen oil. Embryos were viewed on an inverted Leica TCS-SP2 confocal microscope using a 40x oil immersion lens, 488 nm (EGFP) and 543 nm (mRFP) laser lines, and frame-averaging of 4. Images were collected every 20 sec, 30 sec or 40 sec depending on the experiment.

## Results

### Mapping BEAF-BEAF interactions

The carboxy end of BEAF meditates interactions between BEAF proteins [19]. This region contains a putative leucine zipper and a BESS domain [22]. Leucine zippers are well-known dimerization motifs, while the BESS domain of Dip3 (Dorsal-interacting protein 3) is a protein-protein interaction domain [38]. To determine if one or both of these features mediates BEAF-BEAF interactions, we performed yeast 2-hybrid (Y2H) assays and co-immunoprecipitation of bacterially expressed proteins.

Full-length or truncated forms of 32B were fused to the carboxy-side of both the GAL4 DNA binding domain (GAL4-BD) and the GAL4 activation domain (GAL4-AD) (Fig 1A). All combinations were tested in Y2H assays (Table 1), and representative results are shown in Fig 1B with the remaining results shown in S1 Fig. None of the GAL4-BD fusion proteins activated the *ADE2* and *HIS3* reporter genes on their own or together with an empty GAL4-AD. Activation of the reporter genes required that both fusion proteins had the BESS domain. As shown in Fig 1B and S1 Fig, the activation strength for BESS with 32B or LZB (one putative leucine zipper present) is similar to that of 32B with itself, LZB with itself, and 32B with LZB (two putative leucine zippers present) regardless of which protein has the GAL4-BD or GAL4-AD. However, activation was weaker when only the BESS domain was present on both the GAL4-BD and GAL4-AD, indicating that the presence of one putative leucine zipper strengthened the interaction between BESS domains.

Co-immunoprecipitation experiments were performed as another method to examine BEAF-BEAF interactions. Full length 32B and N-terminal FLAG-tagged regions of BEAF, indicated in Fig 2A, were co-expressed in *E*. *coli* from bicistronic operons. We found that co-expression was necessary, interactions were not detected if the proteins were expressed separately and then mixed. Expression of the FLAG-tagged putative leucine zipper was difficult, so sequences were extended about 60 amino acids upstream and several amino acids downstream of the region of interest. Protein extracts were prepared, and immunoprecipitations were performed with anti-FLAG antibody coupled to agarose. Consistent with the Y2H results, Western blot analysis indicated that the BESS domain alone is sufficient to co-precipitate 32B while the putative leucine zipper is not (Fig 2B).

Taken together, we conclude that the BESS domain is required for BEAF-BEAF interactions. However, the presence of at least one putative leucine zipper strengthens the interaction. The putative leucine zipper might contribute either by direct contact or by stabilizing the BESS domain. This effect appears to be asymmetric, since only one putative leucine zipper is required to strengthen the BESS-BESS interaction.

### Testing mutant *BEAF* rescue transgenes

To further characterize BEAF, we tested the ability of deletion mutants of 32B to rescue flies with the null *BEAF*^*AB-KO*^ mutation. We used 32B because when we made the *BEAF*^*AB-KO*^ mutation we found that it is essential, while 32A is not [23]. Comparison of BEAF sequences from 21 *Drosophila* species [39, 40] found that most sequences of the BEAF proteins are highly conserved (S2 Fig). This includes the N-terminal 80 amino acids of 32A and 32B that are derived from different exons and contain different DNA-binding BED fingers [20]. Although only 32B is essential, perhaps 32A enhances fitness of flies outside of a laboratory setting. Because we previously found that BEAF lacking a DNA binding domain functions as a dominant negative [24], we did not delete sequences from this region. The remaining 200 amino acids of 32A and 32B are identical since they are derived from the same exon. Functions for the first 120 amino acids of this region are unknown, although it has been predicted to be phosphorylated and O-linked glycosylated [16, 21]. We refer to this as the Middle region, or M. One region of the BEAF proteins that is not highly conserved encompasses the last 40 amino acids of M, which we refer to as the Middle-NonConserved or M-NC. The C-terminal 80 amino acids have the putative leucine zipper, or LZ, followed by the BESS domain. Based on these considerations, we constructed mutant *32B* genes in which sequences encoding M, M-NC, LZ, or BESS were deleted (Fig 3). To facilitate detection of the proteins in transgenic flies, mRFP was fused to the C-terminus. Because BEAF does not have an identifiable nuclear localization signal, an SV40 NLS was placed at the C-terminus of RFP in case any of the deletions removed the BEAF NLS.

Results of the rescue crosses are presented in Table 2. Two or more transgenic lines were tested for each deletion construct, and five crosses were set up for each line to test for rescue. Deletion of either M or BESS resulted in mutant 32B proteins that were unable to rescue the *BEAF*^*AB-KO*^ allele. Deletion of M gives a more severe phenotype than a complete lack of BEAF, since even the F1 generation failed to produce adults for all crosses. In contrast, a few F1 adults were obtained with the BESS deletion or no rescue transgene. Deletion of LZ provided partial rescue. Two of four transgenic lines gave adults up to the F2 generation (ΔLZ-3B, ΔLZ-3C), although most of these crosses gave 10 or fewer adults in the F1 and F2 generations. This variability is likely due to different expression levels at the different transgene insertion locations. For line ΔLZ-3B, one of five crosses rescued. Development was delayed and not many adults were obtained, but with care it survived over 20 generations. 32B protein lacking M-NC was able to rescue, although there was a developmental delay of a few days from egg to adult. Thus the M region and BESS domain are essential, M-NC is not needed, and lack of the putative leucine zipper results in a hypomorphic 32B protein.

**Table 2:** Test for rescue of the *BEAF*^*AB-KO*^ allele by mutant *32B* transgenes.

			% of crosses surviving to generation:			
Transgene	Line	Crosses	F1	F2	F3	>F3
None	AB-KO	4	75	0	-	-
ΔM	XA	5	0	-	-	-
	3A	5	0	-	-	-
ΔM-NC	3A	5	80	0	-	-
	3B	5	100	100	100	100
	3C	5	100	100	100	100
ΔLZ	3B	5	80	60	40	20
	3C	5	100	20	0	-
	3D	5	0	-	-	-
	3E	5	20	0	-	-
ΔBESS	3A	5	80	0	-	-
	3B	5	20	0	-	-
	3C	5	0	-	-	-
	XA	5	60	0	-	-
	XB	5	60	0	-	-

All fly lines are homozygous for the *BEAF*^*AB-KO*^ allele, with the indicated mutant *BEAF* transgene on the indicated chromosome followed by a letter designating independent transformants. Four or five replicate crosses were set up for each fly line, as indicated. Lines that survived >3 generations survived over 20 generations before the experiment was discontinued.

**Fig 4:**
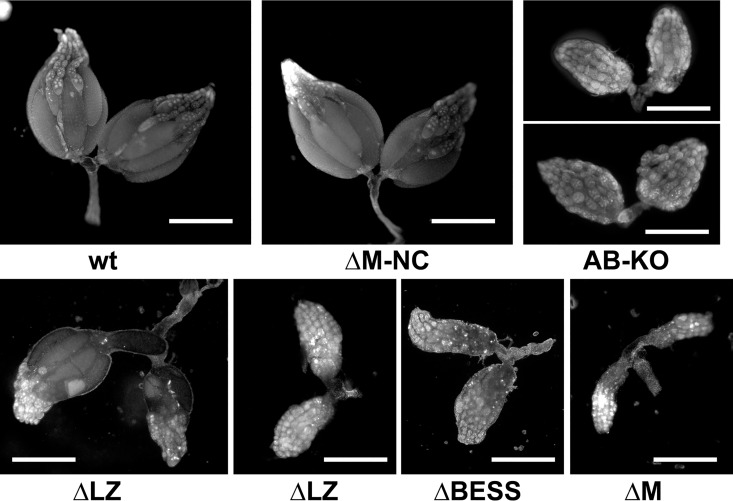
Ovary phenotypes in the presence of various 32B mutations. Females homozygous for the *BEAF*^*AB-KO*^ allele or the indicated *mutant 32B* transgene and the *BEAF*^*AB-KO*^ allele were mated to wild-type males for 3 days before dissecting out ovaries and staining DNA with DAPI. Bars represent 0.5 mm. See text for details.

Ovary development is affected by a lack of BEAF, such that females are nearly sterile [23]. We examined ovaries from flies of the appropriate genotypes and found that the degree of rescue of the *BEAF*^*AB-KO*^ allele by the mutant transgenes correlated with ovary development (Fig 4). Deletion of the BESS domain resulted in underdeveloped ovaries similar to that previously reported for the *BEAF*^*AB-KO*^ allele (60% to 70% of ovaries appear as in Fig 4, while the rest had a couple ovarioles producing a few eggs). Deletion of the M region resulted in more severely underdeveloped ovaries, consistent with the rescue crosses that indicated that 32B lacking M is worse than a lack of 32B (at least 90% of ovaries appear as in Fig 4). Deletion of LZ resulted in underdeveloped ovaries in the ΔLZ-3C, 3D and 3E lines (60% to 80% appear as in the right panel of Fig 4), while in the ΔLZ-3B line that rescued at least one side of most ovaries developed and produced eggs (around 80% appear at least as normal as in the left panel of Fig 4). Fairly normal ovary development occurred when M-NC was deleted (over 90% appear as in Fig 4).

### The dynamics of 32A and 32B during mitosis

**Fig 5:**
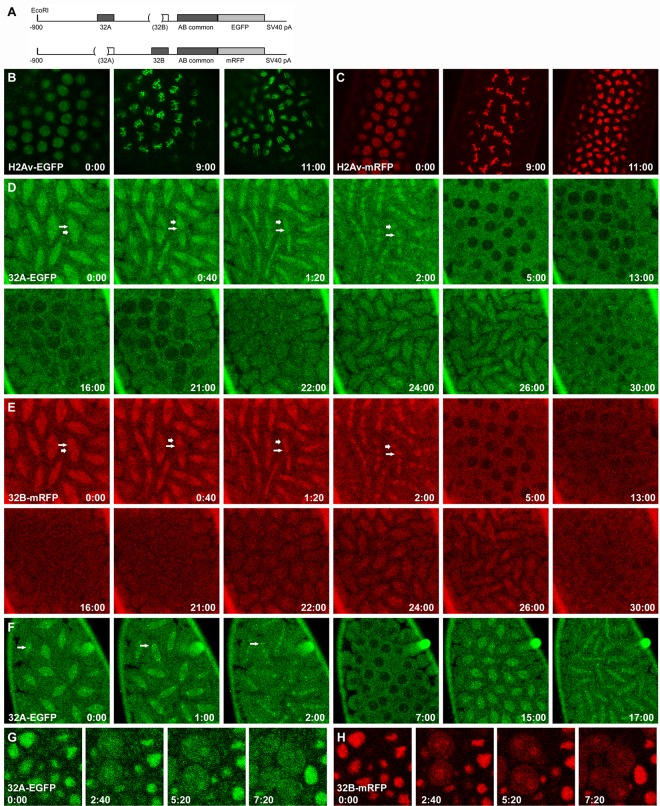
Dynamics of 32A and 32B during mitosis. A. Schematic of the *32A-EGFP* and *32B-mRFP* transgenes. Genomic sequences are present from -900 to the codon for the last amino acid of BEAF, with the indicated sequences deleted so only 32A or 32B can be produced while allowing expression from endogenous *BEAF* sequences. B, C. Dynamics of H2Av-EGFP or H2Av-mRFP, respectively, during interphase and the metaphase and anaphase stages of mitosis in syncytial embryos. See also S1 and S2 Videos. D, E. Time course showing the dynamics of 32A-EGFP or 32B-mRFP, respectively, during two rounds of mitosis in the same syncytial embryo. Short, thick arrows point to chromosomal locations (metaphase at t = 0:00, anaphase to telophase in subsequent panels); longer, thin arrows point to what appears to be the mitotic spindle. See also S3 Video of this embryo and, for other examples, S4–S6 Videos. F. Time course showing the dynamics of 32A-EGFP during two rounds of mitosis in a syncytial embryo. Bright spots of 32A-EGFP that appear to be centromeric are prominent in this embryo (arrows). See also S3 Fig and S4–S6 Videos. G, H. Time course in an embryo during germ band elongation showing the dynamics of 32A-EGFP or 32B-mRFP, respectively, during mitosis. See also S7 Video. All times are in min:sec. Note that all embryos have a wild-type *BEAF* gene, but similar results were obtained in a *BEAF*^*AB-KO*^ background. See text for details.

BEAF was shown to remain bound to mitotic chromosomes [30], while genome-wide mapping found that 32B binds to more sites than does 32A [26]. As part of our characterization of the two BEAF proteins, we decided to investigate their dynamics during mitosis to see if we could confirm their association with mitotic chromosomes and if there were differences between the BEAF isoforms. This was done using live cell microscopy of embryos expressing 32A-EGFP or 32B-mRFP transgenes driven by their endogenous promoters (Fig 5A). The level of expression was sufficient to allow rescue of the null *BEAF*^*AB-KO*^ allele by 32B-mRFP. For comparison, we used histone H2Av transgenes tagged with either EGFP or mRFP. As shown in syncytial embryos, the histone proteins were completely nuclear during interphase and remained on chromosomes throughout mitosis (Fig 5B and 5C and S1 and S2 Videos). This also showed that the levels of background autofluorescence was low in both the green and red channels.

The dynamics of 32A-EGFP and 32B-mRFP were more complex than H2Av and showed differences from each other (Fig 5D–5H and S3–S7 Videos). To directly compare their dynamics, we observed both forms of BEAF in the same embryos. In contrast to H2Av, there was a general background haze. Based on the low background fluorescence with H2Av or with wild-type embryos lacking EGFP and mRFP, as well as the dynamics of the haze during mitosis, this indicated that there is a population of BEAF outside of nuclei. This, combined with low fluorescence intensities, made it difficult to visualize nuclear dynamics with high resolution. Consistent with published results, it appears that 32A and 32B remain on chromosomes during mitosis (for example, short, thick arrows in Fig 5D and 5E panels 0:00–2:00 and S3–S6 Videos), potentially acting as mitotic bookmarks for the next interphase [31]. Chromosomes were difficult to discern the over the background haze and what appears to be BEAF associated with the mitotic spindle (for example, longer, thin arrows in Fig 5D and 5E panels 0:00–2:00 and S3–S6 Videos). This indicates that only subpopulations of the BEAF proteins remain on mitotic chromosomes, similar to findings for other proteins such as TFIID, Polycomb group proteins and mammalian CTCF [41–43]. Consistent with association of 32A and 32B with the mitotic spindle, BEAF was reported to physically interact with Chromator [32]. BEAF and Chromator peaks frequently overlap in genome-wide maps [44], and Chromator reorganizes during mitosis to become part of the mitotic spindle matrix [33]. To explore this in more detail, we observed 32A-EGFP and H2Av-mRFP in the same syncytial embryo (the converse experiment could not be done because the brighter H2Av-EGFP spilled into the red channel and partially obscured the dimmer 32B-mRFP). In agreement with the results described above, we again observed that subpopulations of 32A appear to associate with chromosomes and the mitotic spindle (S3 Fig and S6 Video).

In addition to the unexpected background haze of 32A and 32B, another curious aspect of the dynamics of BEAF is that it is largely excluded from nuclei as they reform in syncytial embryos (for example, Fig 5D and 5E panels 5:00, 13:00 and 30:00). Somewhat similar dynamics have been observed for NONA, Hrb57A, and Rod, although these are not sequence-specific DNA binding proteins [45–47]. To determine if this behavior is limited to early embryogenesis when nuclei rapidly cycle between S and M phases before cellularization takes place, we observed mitosis in later embryos where the cell cycle has distinct G1 and G2 phases. We found that both BEAF proteins are mostly nuclear in interphase cells (Fig 5Gand 5H panel 0:00 and S7 Video), as we also observe for larval and adult tissues. During mitosis, both proteins become largely diffuse with some remaining on chromosomes and possibly the mitotic spindle (Fig 5G and 5H panels 2:40 and 5:20). At the end of mitosis both 32A and 32B are homogenously distributed between the cytoplasm and nuclei, they are not excluded from nuclei as they reform (Fig 5G and 5H panel 7:20). Why BEAF behaves differently in early compared to late embryos is not clear. It could be related to the activation of zygotic transcription during the maternal-to-zygotic transition, and post-translational modifications such as phosphorylation [16] and O-glycosylation [21] could play a role.

In many embryos we observed that 32B entered nuclei earlier than 32A, with 32A entering just prior to prophase (for example, Fig 5D and 5E panels 16:00 and 21:00). However, in some embryos 32B appeared to enter later along with 32A. Another difference occasionally observed was the presence of two to four bright spots of 32A-EGFP during mitosis (Fig 5F and S4 Video). This was never observed for 32B-mRFP. We compared these 32A-EGFP spots to H2Av-mRFP-labeled chromosomes (S3 Fig and S6 Video). The location and dynamics of the bright spots are most consistent with a population of 32A, but not 32B, associating with centromeres or possibly kinetochores. These differences between 32A and 32B are interesting, since both isoforms share the same BEAF-BEAF interaction domain. This suggests that the unique regions of the proteins, which contain their DNA binding domains, influence interactions between, and the dynamics of, 32A and 32B.

## Discussion

To better understand the BEAF proteins, we characterized how BEAF subunits interact with each other and identified essential domains. We found that the BESS domain is necessary and sufficient for interactions between BEAF subunits. It is also essential, since 32B was unable to rescue the null *BEAF*^*AB-KO*^ mutation when it was deleted. The presence of the putative leucine zipper on at least one subunit strengthens the interaction between BESS domains. One possible explanation is that this region helps stabilize the structure of the BESS domain, facilitating stronger BESS-BESS interactions. Another possibility is that the putative leucine zipper is directly involved in an asymmetric interaction with interacting BESS domains. This latter possibility is consistent with results suggesting BEAF can form trimers [19] and possibly larger oligomers (CMH, unpublished). Yet the putative leucine zipper is not essential since 32B lacking it is able to rescue a null mutation. However, this mutant 32B is hypomorphic. Only two of four transgenic *ΔLZ* lines could rescue to the F2 generation, and only one line could be kept alive beyond the F2. Although it could be maintained indefinitely, it was difficult to keep alive and inspection of ovaries indicated there was only a partial rescue of the fecundity defect observed in *BEAF*^*AB-KO*^ flies.

No function is known for the 120 amino acid middle region of BEAF, although this region is thought to be a target for post-translational modification. The first 80 amino acids of this region are well conserved in 21 *Drosophila* species, while the last 40 amino acids are not (S2 Fig). 32B lacking the nonconserved part of the middle region was able to rescue the *BEAF*^*AB-KO*^ mutation. It has been proposed that this region is the major target for O-linked glycosylation, which somehow results in nuclear matrix-association of BEAF [21]. Either other regions can also be glycosylated, or this modification is not essential for BEAF function. On the other hand, deletion of the entire middle region was more lethal than the null *BEAF*^*AB-KO*^ mutation, and the ovary phenotype was more severe. Presumably 32B lacking the middle region interferes with ovary development more than a lack of 32B does due to defects in DNA binding or interactions with partner proteins or both.

BEAF binds DNA in an unusual manner. Most attention has been focused on 32B since it is essential while 32A is not [23], and genome-wide mapping suggests that the 32B binding activity is dominant [26]. The discussion here will also focus on 32B, while pointing out that 32A binds to a different DNA sequence. BEAF was initially purified based on binding to the *scs’* insulator [16], which has two similar binding sites for BEAF. Both binding sites have three copies of the 32B binding motif, CGATA, and evidence indicates that BEAF can bind as a trimer [19]. This and other early work on BEAF [18] led to the model that it binds clusters of three or more CGATA motifs within some window, often somewhat arbitrarily set at 100 bp. But genome-wide mapping of BEAF has not revealed any clear rules on spacing or relative orientation of CGATA motifs in peak regions, and extensive mobility shift assays did not uncover any rules for optimal binding [26]. Some sequences that showed strong binding by bacterially expressed 32B or *Drosophila* nuclear extract BEAF have only 2 CGATA motifs, while others with 3 or more CGATA motifs were only poorly bound. This ambiguity suggests that BEAF has unusual flexibility in its ability to bind CGATA motifs, with the possibility that other DNA sequences might also contribute. One explanation for this could be that the middle region serves as a flexible spacer that allows BEAF subunits interacting via their BESS domains to bind diverse arrangements of CGATA motifs with their BED fingers. Deleting this spacer would have an impact on DNA binding by BEAF complexes, depending on the spacing and orientations between CGATA motifs at specific sites. This defective targeting to DNA might explain the strong phenotype of BEAF lacking the middle region. In this model, shortening the spacer by the 40 amino acid nonconserved region only weakly impacts on the reach of the flexible spacer. This does not exclude other possible functions for the middle region, such as interacting with partner proteins.

The dynamics of BEAF during mitosis were more complex than anticipated. BEAF was observed on mitotic chromosomes of fixed cells [30] and has been mapped on mitotic chromosomes by ChIP-seq [48]. Yet the fraction of fluorescently tagged BEAF on mitotic chromosomes is low enough that the chromosomes cannot be seen with much resolution over the haze of free BEAF. It also appears that some BEAF is associated with the mitotic spindle, perhaps through interactions with Chromator [33]. In syncytial embryos, at the end of mitosis both 32A and 32B are largely excluded from nuclei. 32B often enters nuclei earlier than 32A, with 32A entering just prior to prophase. Another difference between 32A and 32B was that 32A sometimes showed high local concentrations in what appear to be the centromeres or kinetochores of condensed chromosomes. This was never observed for 32B. Later in embryogenesis, BEAF is not excluded from nuclei when they reform but seems to be evenly distributed between the nucleus and cytoplasm. BEAF then accumulates in nuclei so that most BEAF is in interphase nuclei. It is not clear why BEAF behaves differently in early compared to late embryos. It could be related to the maternal-to-zygotic transition. It is also not clear why entry of 32B into nuclei often precedes 32A, especially since they share the same BESS domain. This suggests the unique sequences that include the DNA binding domain can influence BEAF-BEAF interactions. This provides a first glimpse into BEAF dynamics. Additional experiments, for instance with fluorescent-protein tagged microtubules, will be needed to gain more insight into the behavior of BEAF and its significance.

BEAF was initially identified as an insulator-binding protein, and was subsequently found to bind near hundreds of promoters. Understanding how it leads to insulator activity and what its role is at promoters are ongoing challenges. The characterization presented here contributes to our understanding of functional domains in BEAF. It also found that while BEAF might contribute to epigenetic memory through mitotic bookmarking, much BEAF is released from mitotic chromosomes, and some BEAF might play a role in the mitotic spindle matrix. This needs to be taken into consideration in future studies of BEAF. In addition, the differences in dynamics of 32A and 32B in syncytial embryos suggests the unique N-termini with the DNA binding domains influence BESS-mediated interactions between BEAF subunits. Together, these results provide a foundation for future studies into the role of BEAF at promoters and during mitosis.

## Supporting Information

S1 Fig Yeast 2-hybrid assay results for MLZ, LZ and LZB fused to the GAL4 BD. A. Schematic of 32B and parts derived from 32B that were fused at the carboxy ends of the GAL4 DNA-binding domain (BD) and activation domain (AD). Gray rectangle: 32B unique sequences, which encompass the DNA-binding BED finger. First hatched rectangle: putative leucine zipper. Second hatched rectangle: BESS domain. Numbers indicate the first and last amino acid present in the truncated proteins. B. Y2H results for MLZ (top panels), LZ (middle panels) and LZB (bottom panels) fused to the GAL4 BD. Serial 10-fold dilutions of yeast were spotted onto the plates. Left panels (-Trp -Leu) show growth on plates selecting for the presence of the BD and AD plasmids. Right panels (-Trp -Leu -Ade -His) show growth on plates selecting for the presence of the BD and AD plasmids and the expression of two reporter genes. For comparison with Fig 1 and Table 1.
(TIF)

S2 Fig Comparison of BEAF-32A and 32B protein sequences in 21 *Drosophila* species. A. Abbreviations and species used. Species are listed in approximate order of evolutionary distance from *D*. *melanogaster*. B. Boxshade for BEAF-32B. Shown above the boxshade are the BED C_2_H_2_ zinc finger sequence (blue font with potential zinc-coordinating C and H in red); putative leucine zipper sequence (orange font) and BESS domain sequence (aqua font). C. Boxshade of the unique BEAF-32A sequence, plus 10 amino acids shared with BEAF-32B. Shown above the sequence is the BED C_2_H_2_ zinc finger sequence (purple font with potential zinc-coordinating C and H in aqua).
(PDF)

S3 Fig Dynamics of 32A-EGFP and H2Av-mRFP during mitosis in a syncytial embryo. A. Schematic of the *32A-EGFP* and *32B-mRFP* transgenes. Genomic sequences are present from -900 to the codon for the last amino acid of BEAF, with the indicated sequences deleted so only 32A or 32B can be produced while allowing expression from endogenous *BEAF* sequences. B, C. Dynamics of H2Av-mRFP or 32A-EGFP, respectively, in the same syncytial embryo during two rounds of mitosis. Potential association of 32A-EGFP with the mitotic spindle is especially apparent during metaphase, in panels 9:00 and 10:00. Bright spots of 32A-EGFP can be seen at the metaphase plate in the 10:00 panel, and at the spindle pole-proximal tips of the chromosomes in the 11:30 and 12:00 panels, suggesting that some 32A is associated with centromeres or kinetochores. See also S6 Video. All times are in min:sec.
(TIF)

S1 Video Dynamics of histone H2Av-EGFP during mitosis in a syncytial embryo. This embryo is shown in Fig 5B.
(AVI)

S2 Video Dynamics of histone H2Av-mRFP during mitosis in a syncytial embryo. This embryo is shown in Fig 5C.
(AVI)

S3 Video Dynamics of 32A-EGFP and 32B-mRFP during mitosis in a syncytial embryo. This embryo is shown in Fig 5D and 5E.
(MP4)

S4 Video Dynamics of 32A-EGFP during mitosis in a syncytial embryo. This embryo is shown in Fig 5F.
(AVI)

S5 Video Dynamics of 32B-mRFP during mitosis in a syncytial embryo. (AVI)

S6 Video Dynamics of 32A-EGFP and H2Av-mRFP during mitosis in a syncytial embryo. This embryo is shown in S3 Fig.
(AVI)

S7 Video Dynamics of 32A-EGFP and 32B-mRFP during mitosis in an embryo undergoing germ band elongation. This embryo is shown in Fig 5G and 5H.
(AVI)
