# Supplementary figures and images for: Characterization of the Drosophila BEAF-32A and BEAF-32B Insulator Proteins
Source: PLoS One. 2016 Sep 13;11(9):e0162906. doi: 10.1371/journal.pone.0162906 (PMC5021357; doi:10.1371/journal.pone.0162906)

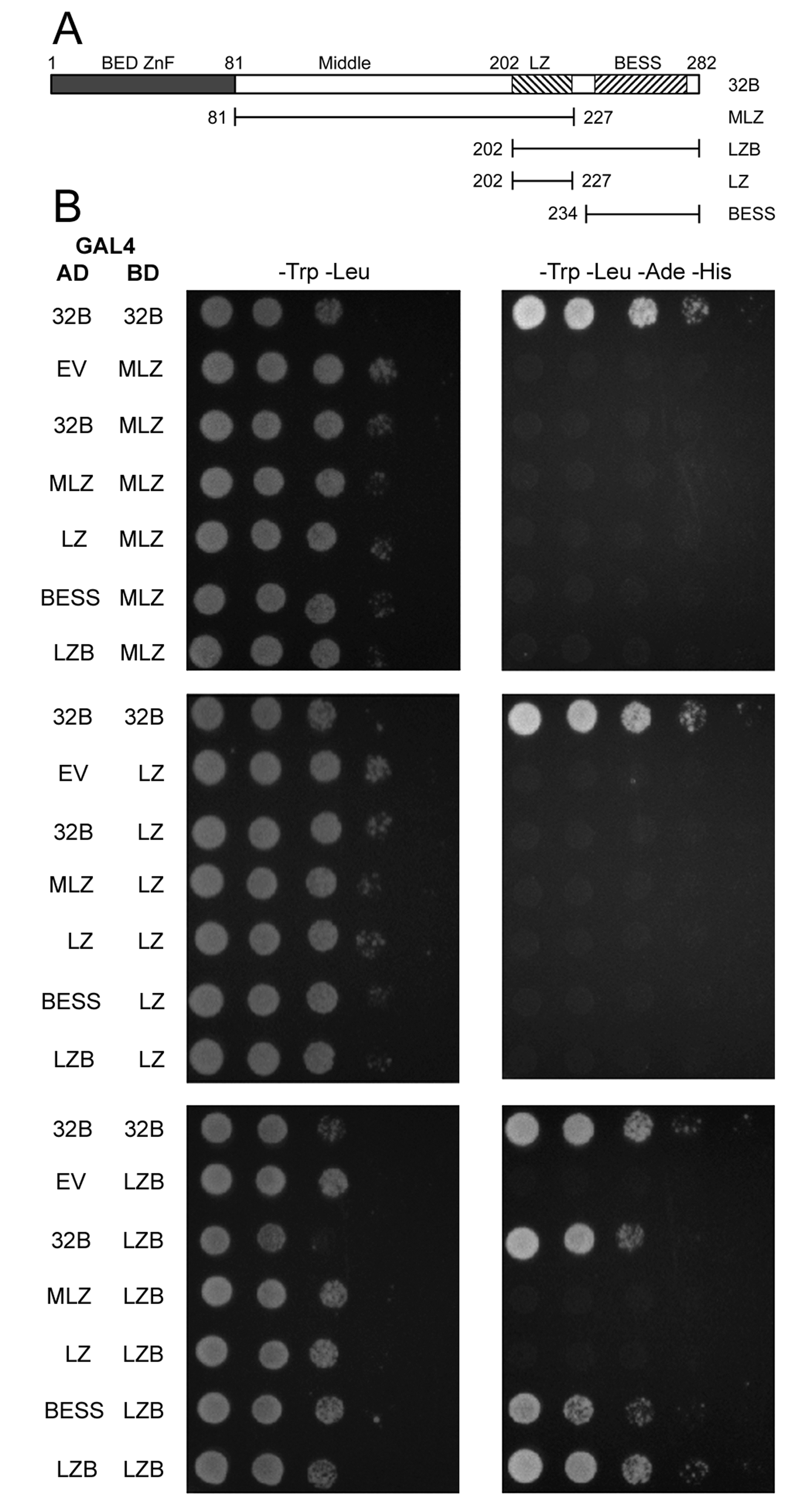

Supplement: S1 Fig — A. Schematic of 32B and parts derived from 32B that were fused at the carboxy ends of the GAL4 DNA-binding domain (BD) and activation domain (AD). Gray rectangle: 32B unique sequences, which encompass the DNA-binding BED finger. First hatched rectangle: putative leucine zipper. Second hatched rectangle: BESS domain. Numbers indicate the first and last amino acid present in the truncated proteins. B. Y2H results for MLZ (top panels), LZ (middle panels) and LZB (bottom panels) fused to the GAL4 BD. Serial 10-fold dilutions of yeast were spotted onto the plates. Left panels (-Trp -Leu) show growth on plates selecting for the presence of the BD and AD plasmids. Right panels (-Trp -Leu -Ade -His) show growth on plates selecting for the presence of the BD and AD plasmids and the expression of two reporter genes. For comparison with Fig 1 and Table 1. (TIF) [file pone.0162906.s001.tif]

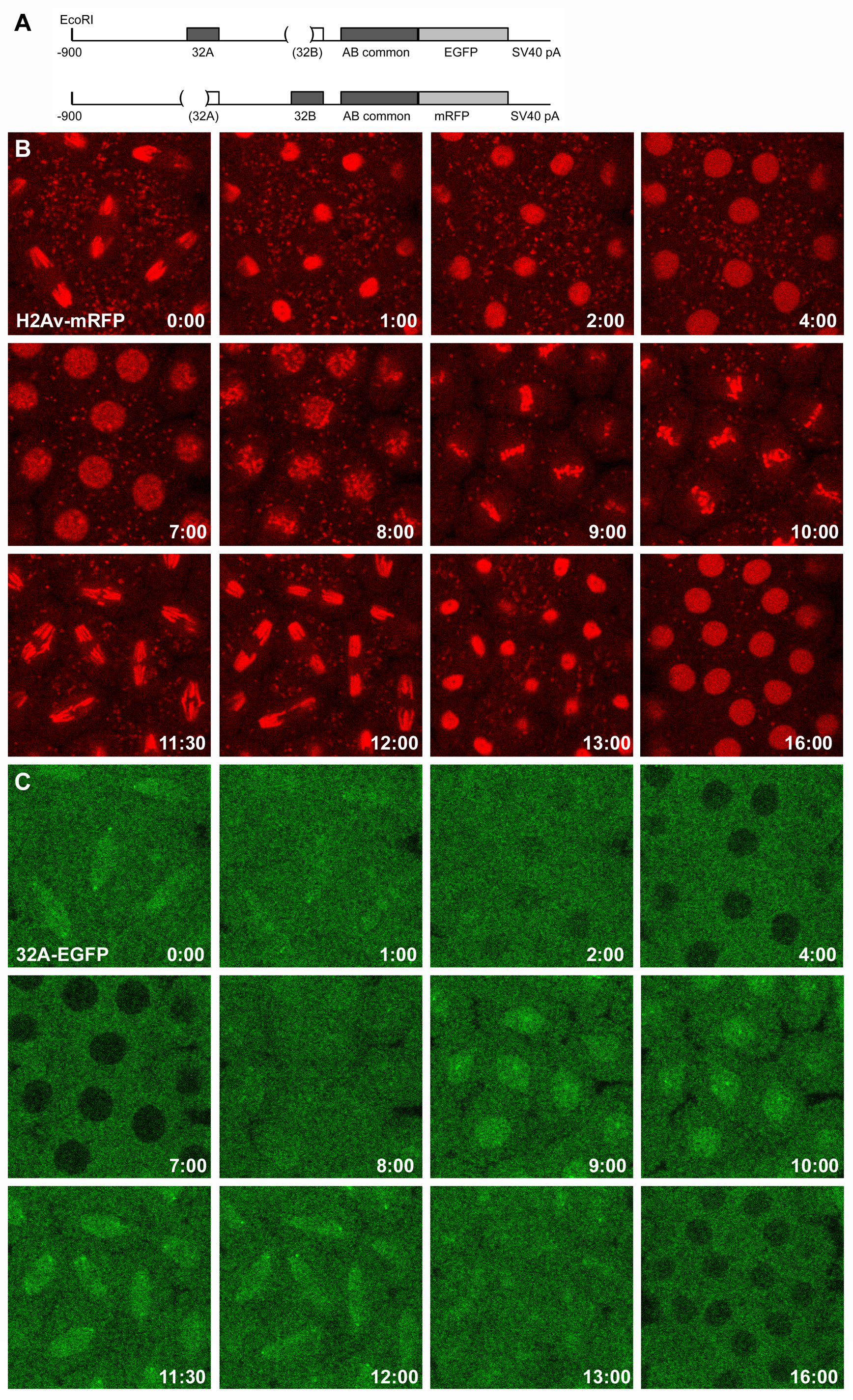

Supplement: S3 Fig — A. Schematic of the 32A-EGFP and 32B-mRFP transgenes. Genomic sequences are present from -900 to the codon for the last amino acid of BEAF, with the indicated sequences deleted so only 32A or 32B can be produced while allowing expression from endogenous BEAF sequences. B, C. Dynamics of H2Av-mRFP or 32A-EGFP, respectively, in the same syncytial embryo during two rounds of mitosis. Potential association of 32A-EGFP with the mitotic spindle is especially apparent during metaphase, in panels 9:00 and 10:00. Bright spots of 32A-EGFP can be seen at the metaphase plate in the 10:00 panel, and at the spindle pole-proximal tips of the chromosomes in the 11:30 and 12:00 panels, suggesting that some 32A is associated with centromeres or kinetochores. See also S6 Video. All times are in min:sec. (TIF) [file pone.0162906.s003.tif]
