# Supplementary material for: Characterization of the Drosophila BEAF-32A and BEAF-32B Insulator Proteins
Source: PLoS One. 2016 Sep 13;11(9):e0162906. doi: 10.1371/journal.pone.0162906 (PMC5021357; doi:10.1371/journal.pone.0162906)
Supplement: S2 Fig — A. Abbreviations and species used. Species are listed in approximate order of evolutionary distance from D. melanogaster. B. Boxshade for BEAF-32B. Shown above the boxshade are the BED C2H2 zinc finger sequence (blue font with potential zinc-coordinating C and H in red); putative leucine zipper sequence (orange font) and BESS domain sequence (aqua font). C. Boxshade of the unique BEAF-32A sequence, plus 10 amino acids shared with BEAF-32B. Shown above the sequence is the BED C2H2 zinc finger sequence (purple font with potential zinc-coordinating C and H in aqua). (PDF) [file pone.0162906.s002.pdf]

Supplemental Figure S2. Boxshade of BEAF protein sequences from 21 *Drosophila* species.

A

D.mel: Drosophila melanogaster

D.sec: Drosophila sechellia

D.sim: Drosophila simulans

D.ere: Drosophila erecta

D.san: Drosophila santomea

D.yak: Drosophila yakuba

D.bia: Drosophila biarmipes

D.eug: Drosophila eugracilis

D.fic: Drosophila ficusphila

D.tak: Drosophila takahashii

D.ele: Drosophila elegans

D.rho: Drosophila rhopaloa

D.kik: Drosophila kikkawai

D.ana: Drosophila ananassae

D.bip: Drosophila bipectinata

D.per: Drosophila persimilis

D.pse: Drosophila pseudoobscura

D.vir: Drosophila virilis

D.gri: Drosophila grimshawi

D.moj: Drosophila mojavensis

D.wil: Drosophila willistoni

B

BEAF-32B

```
                    GRVSNVWQHYDINEECENFAICRYCGNNISRGGMASNLKGNNTTNLWTHLRHKHRDE
D.mel-32B      1 MPKGRVSNVWQHYDINEECENFAICRYCGNNISRGGMASNLKGNNTTNLWTHLRHKHRDE
D.sec-32B      1 MPKGRVSNVWQHYDINEECENFAICRYCGNNISRGGMASNLKGNNTTNLWTHLRHKHRDE
D.sim-32B      1 MPKGRVSNVWQHYDINEECENFAICRYCGNNISRGGMASNLKGNNTTNLWTHLRHKHRDE
D.ere-32B      1 MPKGRVSNVWQHYDINEECENFAICRYCGNNISRGGMASNLKGNNTTNLWTHLRHKHRDE
D.san-32B      1 MPKGRVSNVWQHYDINEECENFAICRYCGNNISRGGMASNLKGNNTTNLWTHLRHKHRDE
D.yak-32B      1 MPKGRVSNVWQHYDINEECENFAICRYCGNNISRGGMASNLKGNNTTNLWTHLRHKHRDE
D.bia-32B      1 MPKGRVSNVWQHYDINEECENFAICRYCGNNISRGGMASNLKGNNTTNLWTHLRHKHSDE
D.eug-32B      1 MPKGRVSNVWQHYDINEECENFAICRYCGNNISRGGMASNLKGNNTTNLWTHLRHKHSDE
D.fic-32B      1 MPKGRVSNVWQHYDINEECENFAICRYCGNNISRGGMASNLKGNNTTNLWTHLRHKHSDE
D.tak-32B      1 MPKGRVSNVWQHYDINEECENFAICRYCGNNISRGGMASNLKGNNTTNLWTHLRHKHSDE
D.ele-32B      1 MPKGRVSNVWQHYDINEECENFAICRYCGNNISRGGMASNLKGNNTTNLWTHLRHKHSDE
D.rho-32B      1 MPKGRVSNVWQHYDINEECENFAICRYCGNNISRGGMASNLKGNNTTNLWTHLRHKHSDE
D.kik-32B      1 MPKGRVSNVWQHYDINEECENFAICRYCGNNISRGGMASNLKGNNTTNLWTHLRHKHSDE
D.ana-32B      1 MPKGRVSSVWQHYDINEECENFAICRYCGNNISRGGMASNLKGNNTTNLWTHLRHKHSDQ
D.bip-32B      1 MPKGRVSSVWQHYDINEECENFAICRYCGNNISRGGMASNLKGNNTTNLWTHLRHKHSDQ
D.per-32B      1 MPKGRVSNVWQHYDINEDCENFAICRYCGNNISRGGMASNLKGNNTTNLWTHLRHKHSDE
D.pse-32B      1 MPKGRVSNVWQHYDINEDCENFAICRYCGNNISRGGMASNLKGNNTTNLWTHLRHKHSDE
D.vir-32B      1 MPKGRVSSVWQHYDINEECENFAVCRYCGNNISRGGMASNLKGNNTTNLWTHLRHKHADE
D.gri-32B      1 MPKGRVSSVWQHYDINEECENFAVCRYCGNNISRGGMASNLKGNNTTNLWTHLRHKHSDE
D.moj-32B      1 MPKGRVSSVWQHYDINEECENFAVCRYCGNNISRGGMASNLKGNNTTNLWTHLRHKHADE
D.wil-32B      1 MPKVRISSVWQHYDVNEECENFAICRYCGNNISRGGMSSNLKGNNTTNLWTHLRHKHSDE
                 *** *:*.******:**:*****:*************:******************* *:
```

```
D.mel-32B    61 VLVSPVQQRPQARMIPIIKK-DKTVRITKAKTLREPLRVAKPKIE--SNVANYLGEAGAL
D.sec-32B    61 VLVSPVQQRPQARMIPIIKK-DKTVRITKAKTLREPLRVAKPKIE--SNVASYLGEAGAL
D.sim-32B    61 VLVSPVQQRPQARMIPIIKK-DKTVRITKAKTLREPLRVAKPKIE--SNVASYLGEAGAL
D.ere-32B    61 VLVSPVQQRPQARMIPIIKK-DKTVRITKAKTLREPLRVAKPKIE--SNVANYLGEAGAL
D.san-32B    61 VLVSPVQQRPQARMIPIIKK-DKTVRITKAKTLREPLRVAKPKID--SNVASYLGEAGAL
D.yak-32B    61 VLVSPVQQRPQARMIPIIKK-DKTVRITKAKTLREPLRVAKPKID--SNVASYLGEAGAL
D.bia-32B    61 VLVSPVQQRSQARMIPIIKK-DKTVRITKAKTLREPLRVAKPKIE--TNVASYLGESGAL
D.eug-32B    61 VLVSPVQQRTQSRMIPIIKK-EKTVRITKAKTLREPLRVAKPKIE--SNVASYLGEAGAL
D.fic-32B    61 VLVSPVQQRPQARMIPIIKK-EKTVRITKAKTLREPLRVAKPKID--SSTANYLGEAGAL
D.tak-32B    61 VLVSPVQQRPQARMIPIIKK-EKTVRITKAKTLREPLRVAKPKIE--SNVASYLGESGAL
D.ele-32B    61 VLVSPVQQRTPTRMIPIIKK-EKTVRITKAKTVREPLRVAKPKID--SSVASYLGEAGAL
D.rho-32B    61 VLVSPVQQRTPSRIIPIIKK-EKTVRITKAKTLREPLRVAKPKIE--SGVASYLGEAGAL
D.kik-32B    61 VLVSPVQQRP-NRVFPIIKK-EKTVRIAKAKQLREPLRLAKPKID-VSG--SYVGETGAL
D.ana-32B    61 VLVSPVQQKS-PRVIPIIKK-EKTVRITKAKQLREPLRLAKPKIEPASGSFIVATESGAL
D.bip-32B    61 VLVSPVQQKS-PRVIPIIKK-DKAVRITKAKQLREPLRLAKPKIEPNSGSFIVATESGAL
D.per-32B    61 VVVNPVQSRQ-GRLIPILKK-EKTVRITKAKQLREPLHVAKPKIERQST--SFVAETGAL
D.pse-32B    61 VVVNPVQSRQ-GRIIPILKK-EKTVRITKAKQLREPLHVAKPKIERQST--SFVAETGAL
D.vir-32B    61 VLVNPIQQRPISRPAVITLKKEKTSRIAKAKVVREPLRVAKPKVD----VHASIA--GAL
D.gri-32B    61 VIVNPIQQRPLSRNAVITLKKEKTSRIAKAKVVREPIRVAKPKID----VHASIT--GAL
D.moj-32B    61 VLVNPIQQRPVSRPAVITLKKEKTSRIAKAKVVREPLRVAKPKID----VHASIS--GAL
D.wil-32B    61 VVVSPIQSQKPRTPVTIKKE--KTVRITKAKQSLPSLRVAKPKVS-LEANTSSASDVGTL
                *:*.*:*.:       *  :  *: **:***    .:::****::            *:*

D.mel-32B   118 PQKWEEYEQNDEISEDIKDIIYSEDPLCYSPIHVMDDEG-LDQPEKQVTVLTHSTSPAGG
D.sec-32B   118 PQKWEEYEQNDEISEDIKDIIYSEDPLCYSPIHVMDDEG-LDQPEKQVTVLTHSTSPAGG
D.sim-32B   118 PQKWEEYEQNDEISEDIKDIIYSEDPLCYSPIHVMDDEG-LDQPEKQVTVLTHSTSPAGG
D.ere-32B   118 PQKWEEYEQNDEISEDIKDIIYSEDPLCYSPIHVMEDDG-LDQPEKQVTVLTHSTSSVGG
D.san-32B   118 PQKWEEYEQNDEISEDIKDIIYSEDPLCYSPIHVMEDEG-LDQPEKQVTVLTHSTSSAGG
D.yak-32B   118 PQKWEEYEQNDEISEDIKDIIYSEDPLCYSPIHVMEDEG-LDQPEKQVTVLTHSTSSAGG
D.bia-32B   118 PQKWEEYEQNDEIGEDIKDIIYSEDPLCYSPIHVMEDEG-LEQGEKQVTVVTHSSGSGAG
D.eug-32B   118 PQKWEEYEQNDEISEDIKDIIYSEDPLCYSPIHVMEDEA-LEQGEKQVTVLSHSSATGGG
D.fic-32B   118 PQKWEEYEQNDEISEDIKDIIYSEDPLCYSPIHVMEDEG-LEQGEKQVTVLSHTGASASG
D.tak-32B   118 PQKWEEYEQNDEIGEDIKDIIYSEDPLCYSPIHVMEDEHGLEHGEKQVTLISGSTATAAG
D.ele-32B   118 PQKWEEYEQNDEIGEDIKDIIYSEDPLCYSPIHVMDEEGGVE---EKQVTVLSHSGAGGG
D.rho-32B   118 PQKWEEYEQNDEISEDIKDIIYSEDPLCYSPIHVMDEEGGVEHAQEKQVTVLSHSSAAGG
D.kik-32B   116 PQKWEEYEQNDEITDDIKDIIYSEDPLCYSPIHVMEDET---LETGEKVHVVGNASVVGA
D.ana-32B   119 PQKWEEYEHTDEISEDIKDIIYSEDPLCYSPIHVMEDEAYETGDKVTVLNATGTSSSGGR
D.bip-32B   119 PQKWEEYEQTDEISEDIKDIIYSEDPLCYSPIHVMEDEAFEAGDKVTVLNTTGTSGTGGR
D.per-32B   117 PQKWEEYEQNDGISEDIKDIIYNEDPLCYSPIHVMEEEVALESGEKVTVLNPS-------
D.pse-32B   117 PQKWEEYEQNDGISEDIKDIIYSEDPLCYSPMHVMEEEVALESGEKVTVLNPS-------
D.vir-32B   115 PQKWEEYEQNDEVSEDIKDIIYSEDPLCYSPIQVVEDEMLDAGEKVTVVSS--NRTTTNP
D.gri-32B   115 PQKWEEYEQNEEVSEDIKDIIYSEDPLCYSPIQVVEDEMLESGEKVTLMSSGSNRTTTVP
D.moj-32B   115 PQKWEEYEQNDEVAEDIKDIIYNEDPLCYSPIQVVEDEMLDTGEKVTVMTSN-RVSSANP
D.wil-32B   118 PQKWEDYDQGDEIG-DIKDIIYNEDPLCYSPIQVVEEEMIDTSEKVTTVLNPTSSISRSQ
                *****:*:: : :  *******.********::*::::
```

```
                                          AKQLKNNLETSIERLTAVSEQLSYII
D.mel-32B  177 SSRPIGVGS--GVQATVVASTSSSSSSAKQLKNNLETSIERLTAVSEQLSYIIQQNHE-E
D.sec-32B  177 SSRAIGVGS--GVQATVVASTSSSSSSAKQLKNNLETSIERLTAVSEQLSYIIQQNHE-E
D.sim-32B  177 SSRPIAVGS--GVQATVVASTSSSSSSAKQLKNNLETSIERLTAVSEQLSYIIQQNHE-E
D.ere-32B  177 SSRPSAVGS--GAQATVVASTSSSNSSAKQLKNNLETSIDRLTTVSEQLSYIIQQNQE-E
D.san-32B  177 SSRPSAVGS--GVQATVVASTSSSNNSAKQLKNNLETSIDRLTTVSEQLSYIIQQNHE-E
D.yak-32B  177 SSRPSAVGS--GVQATVVASTSSSNNSAKQLKNNLETSIDRLTTVSEQLSYIIQQNHE-E
D.bia-32B  177 SSRPSAASG--VQATVVASSSTATNNSAKQLKNNLETSIDRLTTVSEQLSYIIQQNHE-E
D.eug-32B  177 SSRPSAAAQ--GQATVVASTSGSN--AAKQLKNNLETSIDRLTTVSEQLSYIIQQNHE-E
D.fic-32B  177 GSSRPSAAI--PVVQATVLASTSNNNSAKQLKNNLETSIDRLTTVSEQLGYIIQQNHE-E
D.tak-32B  178 GS-RQSAGG--STVQATVVASTSTNNSAKQLKNNLETSIDRLTTVSEQLSYIIQQNHE-E
D.ele-32B  175 SGSRANAAASQGIQATVVASTSSNNNSAKQLKNNLETSIDRLTTVSEQLSYIIQQNHE-E
D.rho-32B  178 SSSRTSTAGPQVVQATVVASTSANN-SAKQLKNNLETSIDRLTTVSEQLSYIIQQNHE-E
D.kik-32B  173 GGSRASATP----VVATVVASSSASSPAKQLKNNLETSIDRLTTVSEQLSYIIQQSHE-E
D.ana-32B  179 ASVSAPVVAT---------VVATPTNSAKQLKNNLETSIDRLTTVSEQLSYIIQQNHE-E
D.bip-32B  179 ASVSAPVVAT---------VVAAPNNSAKQLKNNLETSIDRLTTVSEQLSYIIQQNHE-E
D.per-32B  170 -GHSRATISSAPVVATVVASNTNTANAAKQLKNNLETSIDRLTTVSEQLSYIIQQSHE-E
D.pse-32B  170 -GHSRATISSAPVVATVVASNTNTANAAKQLKNNLETSIDRLTTVSEQLSYIIQHSHE-E
D.vir-32B  173 VVATVVSAP---------------TSAAKTLKNNLETSIERLTTVSEQLSYVIQQQQEEQ
D.gri-32B  175 VVATVVSGT---------------TSAAKTLKNNLETSIERLTTVSEQLSYVIQQQQE-Q
D.moj-32B  174 VVATVVSAP---------------TSAAKTLKNNLETSIDRLTTVSEQLSYIIQQQQE-E
D.wil-32B  177 SASASVTPMV------TTMVSTSSSNSAKSLKNNLEQSIDRLTSVSEQLTYIIQQNND-E
                                         .** ****** **:***:***** *:**:.:: :
```

```
                 KDDDYYFALSLVPAMRHLSLSRKMYVRSKIQDILFKESED
D.mel-32B  234 LTKDDDYYFALSLVPAMRHLSLSRKMYVRSKIQDILFKESEDSTLAKDE--
D.sec-32B  234 LTKDDDYYFALSLVPVMRHLSLSRKMYVRSKIQDILFKESEDSPVAKDE--
D.sim-32B  234 LTKDDDYYFALSLVPVMRHLSLSRKMYVRSKIQDILFKESEDSPVAKDE--
D.ere-32B  234 LNKDDDYYFALSLVPVMRHLSLSRKMYVRSKIQEILFKESEDSAPAKDE--
D.san-32B  234 HTKDDDYYFALSLVPVMRHLSLSRKMYVRSKIQEILFKESEDSLPAKDE--
D.yak-32B  234 HTKDDDYYFALSLVPVMRHLSLSRKMYVRSKIQEILFKESEDSLPAKDE--
D.bia-32B  234 ATKDDDYYFALSLVPVMRHLSLSRKMYVRSKIQEILFKESEDSVPSKDE--
D.eug-32B  232 QTKDDDYYFALSLVPMMRHLSLSRKMYVRAKIQEILFKESEDGAPTKDE--
D.fic-32B  234 HTKDDDYYFALSLVPVMRHLSLNRKMYVRSKIQEILFKESEDSAPAKDE--
D.tak-32B  234 HTKDDDYYFALSLVPVMRQLSLNRKMYVRSKIQELLFKESEDSAPAKDE--
D.ele-32B  234 QTKDDDYYFALSLVPVMRHLSLNRKLYVRSKIQEILFKESEESTPAKDE--
D.rho-32B  236 HTKDDDYYFALSLVPVMRHLSLNRKLYVRSKIQEILFKESEDSVPAKDE--
D.kik-32B  228 QTKDDDYYFALSLVPIMRHLSINRKMYVRSKIQELLFKESEGESLASKDE-
D.ana-32B  229 LSKDDDYYFALSLVPIMRQLSLSRKLYVRSKIQEILFKESEDPIPSKED--
D.bip-32B  229 LVKDDDYFFALSLVPVMRQLSLTRKLFVRSMITELLYKEAQEPTPPASKEE
D.per-32B  228 HSKDDDYYFALSLVPSMRQMSLSRKLYVRTKIQEVIFKTSEEATSGKDE--
D.pse-32B  228 HIKDDDYYFALSLVPSMRQMSLSRKLYVRTKIQEVIFKTSEEATSGKDE--
D.vir-32B  218 QLKDDDYYFALSLVPIMRQLSVSRKLYVRSKIHEILYKESEETRTKTE---
D.gri-32B  219 QLKDDDYYFALSLVPVMRQLSVSRKMYVRSKIHELLYKESEETTTKIE---
D.moj-32B  218 QIKDDDYYFAMSLVSSMRQLSVNRKLFVRAKIHEILYKENEEAATKAE---
D.wil-32B  230 HSKDDDYYFALSLVPIMRQLSLNRKLYVRSKIQEILFKESEANPSTKQE--
                 *****:**:***. **::*:.**::**: * ::::*  :      .
```

C

BEAF-32A

(only the unique 32A sequences plus 10 shared amino acids [DKTVRITKAK] are shown)

```
                                                         NTKSCVWRFFNLVQCDDHIEPYACCKTCGDLLSY
D.mel-32A    1 MHVEKKSELRSLLKSGDKRCKLVEPRNTKSCVWRFFNLVQCDDHIEPYACCKTCGDLLSY
D.sec-32A    1 MHVEKKSELRSLLKSGDKRCKLVEPRNTKSCVWRFFNLVQCDDHIEPYACCKTCGDLLSY
D.sim-32A    1 MHVEKKSELRSLLKSGDKRCKLVEPRNTKSCVWRFFNLVQCDDHIEPYACCKTCGDLLSY
D.ere-32A    1 MHVEKKSELRSLLKSGDKRCKLVEPRNTKSCVWRFFNLVQCDDHIEPYACCKTCGDLLSY
D.san-32A    1 MHVEKKSELRSLLKSGDKRCKLVEPRNTKSCVWRFFNLVQCDDHIEPYACCKTCGDLLSY
D.yak-32A    1 MHVEKKSELRSLLKSGDKRCKLVEPRNTKSCVWRFFNLVQCDDHIEPYACCKTCGDLLSY
D.bia-32A    1 MHVEKKSELRSLLKSGDKRCKLVEPRNTKSCVWRFFNLVQCDDRIEPYACCKTCGDLLSY
D.eug-32A    1 MHVEKKSELRSLLKSGDKRCKLVEPRNTKSCVWRFFNLVQCDDHIEPYACCKTCGDLLSY
D.fic-32A    1 MHVEKKSELRSLLKSGDKRCKLVEPRNTKSCVWRFFNLVQCDDRIEPYACCKTCGDLLSY
D.tak-32A    1 MHVEKKSELRSLLKSGDKRCKLVEPRNTKSCVWRFFNLVQCDDHIEPYACCKTCGDLLSY
D.ele-32A    1 MHVEKKSELRSLLKSGDKRCKLVEPRNTKSCVWRFFNLVQCDDRIEPYACCKTCGDLLSY
D.rho-32A    1 MHVEKKSELRSLLKSGDKRCKLVEPRNTKSCVWRFFNLVQCDDRIEPYACCKTCGDLLSY
D.kik-32A    1 MHVERKSELRSMLKSGDKRCKLVEPRNTKSCVWRFFNLVSCDDQIEPYACCKTCGDLLSY
D.ana-32A    1 MHVEKKSELRSLLKSGDSRCKLVEPRNTKSCVWRFFNLVSCDDRIEPYACCKTCGDLLSY
D.bip-32A    1 MHVEKKSELRSLLKSGDSRCKLVEPRNTKSCVWRFFNLVSCDDRIEPYACCKTCGDLLSY
D.per-32A    1 MHVEKKSELRSLLKSGDKRCKLVEPRNTKSCVWRFFNLVSCDDKIEPYACCKTCGDLLSY
D.pse-32A    1 MHVEKKSELRSLLKSGDKRCKLVEPRNTKSCVWRFFNLVSCDDKIEPYACCKTCGDLLSY
D.vir-32A    1 MHVEKKSELRSLLKSGDKRCKLVEPRNAKSCVWRFFNLVSCDDRIEPYACCKTCGDLLSY
D.gri-32A    1 MHVEKKSELRSLLKSGDKRCKLVEPRNAKSCVWRFFNLVSCDDRIEPYACCKTCGDLLSY
D.moj-32A    1 MHVEKKSELRSLLKSGDKRCKLVEPRNAKSCVWRFFNLVSCDDRIEPYACCKTCGDLLSY
D.wil-32A    1 MHVEKKSELRSLLKSGDKRCKLVEPRNTKSCVWRFFNLVSCDDRIEPYACCKTCGDLLSY
               ****:******:*****.*********:***********.***:****************

               SGKTGTGSLLRHRCLHSSS
D.mel-32A   61 SGKTGTGSLLRHRCLHSSSS--NDKTVRITKAK
D.sec-32A   61 SGKTGTGSLLRHRCLHSSSS--NDKTVRITKAK
D.sim-32A   61 SGKTGTGSLLRHRCLHSSSS--NDKTVRITKAK
D.ere-32A   61 SGKTGTGSLLRHRCLHSSSS--NDKTVRITKAK
D.san-32A   61 SGKTGTGSLLRHRCLHSSSSS-NDKTVRITKAK
D.yak-32A   61 SGKTGTGSLLRHRCLHSSSSS-NDKTVRITKAK
D.bia-32A   61 SGKTGTGSLLRHRCLH-SNSN---KTVRITKAK
D.eug-32A   61 SGKTGTGSLLRHRCLH-SSSS-NEKTVRITKAK
D.fic-32A   61 SGKTGTGSLLRHRCLHSSSSSSNEKTVRITKAK
D.tak-32A   61 SGKTGTGSLLRHRCLHSNSS--NEKTVRITKAK
D.ele-32A   61 SGKTGTGSLLRHRCLHSSSS--NEKTVRITKAK
D.rho-32A   61 SGKTGTGSLLRHRCLHSSSS--NEKTVRITKAK
D.kik-32A   61 SGKTGTGSLLRHRCLHSSNEK----TVRIAKAK
D.ana-32A   61 SGKTGTGSLLRHRCLHSTPS--NEKTVRITKAK
D.bip-32A   61 SGKTGTGSLLRHRCLHSTPT--NDKAVRITKAK
D.per-32A   61 SGKTGTGSLLRHRCLHSNSNN--EKTVRITKAK
D.pse-32A   61 SGKTGTGSLLRHRCLHSNSNN--EKTVRITKAK
D.vir-32A   61 SGKTGTGSLLRHRCLHTT----NEKTSRIAKAK
D.gri-32A   61 SGKTGTGSLLRHRCLHTT----NEKTSRIAKAK
D.moj-32A   61 SGKTGTGSLLRHRCLHTT----NEKTSRIAKAK
D.wil-32A   61 SGKTGTGSLLRHRCLHTPHN---EKTVRITKAK
               ****************         :  **:***
```

Supplemental Figure S2. Boxshade of BEAF protein sequences from 21 *Drosophila* species. A) Abbreviations and species used. Species are listed in approximate order of evolutionary distance from *D. melanogaster*. B) Boxshade for BEAF-32B. Shown above the boxshade are the BED C_2H_2 zinc finger sequence (blue font with potential zinc-coordinating C and H in red); putative leucine zipper sequence (orange font) and BESS domain sequence (aqua font). C) Boxshade of the unique BEAF-32A sequence, plus 10 amino acids shared with BEAF-32B. Shown above the sequence is the BED C_2H_2 zinc finger sequence (purple font with potential zinc-coordinating C and H in aqua).
